# Institutional pioneers in world politics: Regional institution building and the influence of the European Union

**DOI:** 10.1177/1354066116674261

**Published:** 2016-11-09

**Authors:** Tobias Lenz, Alexandr Burilkov

**Affiliations:** University of Goettingen, Germany; GIGA German Institute of Global and Area Studies, Hamburg, Germany; GIGA German Institute of Global and Area Studies, Hamburg, Germany

**Keywords:** Delegation, diffusion, institutional change, institutional design, regional international organizations, regionalism

## Abstract

What drives processes of institution building within regional international organizations? We challenge those established theories of regionalism, and of institutionalized cooperation more broadly, that treat different organizations as independent phenomena whose evolution is conditioned primarily by internal causal factors. Developing the basic premise of ‘diffusion theory’ — meaning that decision-making is interdependent across organizations — we argue that institutional pioneers, and specifically the European Union, shape regional institution-building processes in a number of discernible ways. We then hypothesize two pathways — active and passive — of European Union influence, and stipulate an endogenous capacity for institutional change as a key scope condition for their operation. Drawing on a new and original data set on the institutional design of 34 regional international organizations in the period from 1950 to 2010, the article finds that: (1) both the intensity of a regional international organization’s structured interaction with the European Union (active influence) and the European Union’s own level of delegation (passive influence) are associated with higher levels of delegation within other regional international organizations; (2) passive European Union influence exerts a larger overall substantive effect than active European Union influence does; and (3) these effects are strongest among those regional international organizations that are based on founding contracts containing open-ended commitments. These findings indicate that the creation and subsequent institutional evolution of the European Union has made a difference to the evolution of institutions in regional international organizations elsewhere, thereby suggesting that existing theories of regionalism are insufficiently able to account for processes of institution building in such contexts.

## Introduction

In 2000, African heads of state strengthened the main framework for institutionalized cooperation on the continent by replacing the Organization of African Unity with the African Union. This transition marked a significant evolution towards more powerful regional institutions, resembling those of the European Union (EU). The creation of a Commission with a codified right to initiate legislation and to bring infringement cases to a new African Court of Justice or Pan-African Parliament led many observers to comment on the apparent ‘organisational mirroring’ occurring between the two bodies (Haastrup, 2013: 789).

This episode poses an important theoretical question: which factors drive processes of institution building within regional international organizations (RIOs)? More specifically, what role does the EU play in them? Most theories of regionalism, and of international cooperation more broadly, are ill-equipped to capture such ‘outside-in’ influences because they locate the main drivers of institution building *within* each respective region. They view institutions primarily as reflecting the processes and structures of a given region, ones that operate from the ‘inside out’. Dominant functional theories of cooperation — such as neofunctionalism (Haas, 1961), (liberal) intergovernmentalism (Moravcsik, 1998) and neoliberal institutionalism (Keohane, 1984; Koremenos et al., 2001) — view RIOs primarily as a response to conflicts or problems of collective action resulting from economic or security-related interdependence within a particular region. As patterns of interdependence shift, organizations change their form.

Similarly, constructivist or transactionalist approaches emphasize the role of communication and collective identities (Adler and Barnett, 1998; Deutsch, 1957; Katzenstein, 2005). They posit that organizations develop in response to changing social processes and structures. Taken together, these benchmark studies see factors *endogenous* to the region as being the drivers of institution building. As a recent review of two key works on the subject perceptively notes: ‘Neither volume tells us much about interregional flows … or about emulation and learning, including the demonstration effects of one type of regionalism on another’ (Acharya, 2007: 637). With their focus on intraregional influences, most such studies treat, in sum, different RIOs as atomistic entities that develop largely independently of each other.

We challenge this widespread assumption in the literature on regionalism by developing a diffusion account of EU influence on other RIOs, then subjecting it to the first systematic large-N analysis thereof. We build on a growing body of research in Comparative Politics and International Relations which posits that units of analysis — be they policies, national institutions or international organizations — need to be conceived of as affecting each other (Braun and Gilardi, 2006; Grigorescu, 2010; Simmons et al., 2006). Institutional choices in some organizations systematically shape institutional choices in others. Diffusion studies thus analyse how ‘decisions in one country [are] influenced … by the ideas, norms, and policies displayed or even promoted by other countries and international organizations’ (Gilardi, 2012: 453).

Developing this premise, we posit that the most prominent institutional pioneer in regionalism, the EU, systematically affects the institutional evolution of RIOs in other parts of the world; we furthermore identify the conditions under which it does so. Arguments about EU diffusion — although occurring under different labels — are long-standing. Early scholars of regionalism acknowledged, but failed to analyse systematically, the possibility of interregional influences (Haas, 1961). Today, claims about the influence that the EU ‘model’ has are commonplace in the literature on regionalism. Fioramonti and Mattheis (2015: 1) suggest, for example, that ‘there is little doubt that the proactive role played by EU institutions to support regionalism has led to a “diffusion” of norms and institutional models’. However, none of these such works present clear propositions about EU influence, nor do they systematically test for its effects. A growing body of case-study work demonstrates such diffusion processes between the EU and other RIOs (Jetschke, 2009; Lenz, 2012; Rüland, 2014). A recent special issue on the topic confidently claims that the impact of EU diffusion is ‘certainly not spurious’ (Börzel and Risse, 2012: 194). While this literature has provided empirically convincing evidence that EU diffusion does indeed affect other RIOs in *some* cases, the external validity of these claims remains uncertain at present. Moreover, while we have an emerging sense of the mechanisms that underlie EU influence, our knowledge about the conditions under which it is likely to matter is limited.

This article offers the first systematic attempt to gauge the effects of the EU on other RIOs by drawing on a new and original data set that measures variation in the institutional design of 34 such organizations on an annual basis in the period from 1950 to 2010. Institutional design has not only become a vibrant field of study in International Relations (Koremenos et al., 2001); evidence is also accumulating that design matters for substantive outcomes in world politics (Koremenos, 2016).^1^ It is also widely seen as one of the most important ‘objects’ of EU influence. Even though EU influence might affect other aspects of regional integration — such as a counterpart’s policy portfolio, the development of their economic integration process or their democratic interventions (Gray, 2014b; Jupille et al., 2013) — formal institutions are nonetheless a key dimension of external influence. Indeed, the EU’s active promotion of regionalism specifically seeks to strengthen regional institutions. Simultaneously, the bulk of the literature concerned with processes of emulation of the EU also examines formal institutions.

We draw on the diffusion literature to theorize two pathways of EU influence: first, its active promotion of regional institution building through financial incentives and structured interaction (active influence); and, second, its provision of institutional designs that serve as a reference point for policymakers in RIOs elsewhere (passive influence). We use prominent diffusion mechanisms as heuristic devices to theorize these two pathways, but our goal is not to adjudicate between different causal mechanisms. Recent research shows that such attempts have proven difficult in quantitative studies due to the difficulty of matching indicators and concepts — and of dealing with the problem that distinct mechanisms ‘are often interrelated’ empirically (Graham et al., 2013: 695; Maggetti and Gilardi, 2016).

Therefore, our analysis focuses rather on delineating and measuring the conditions under which the two identified general pathways of EU influence are likely to matter, and on assessing their relative explanatory power. We also hypothesize that an endogenous capacity for institutional change in RIOs — contractual incompleteness — conditions EU influence. Using fixed-effects panel estimation techniques, our results show that: (1) both the intensity of an RIO’s structured interaction with the EU (active influence) and the EU’s own level of delegation (passive influence) are correlated with the level of delegation within other RIOs; (2) passive EU influence exerts a larger overall substantive effect than does active EU influence; and (3) these effects are strongest among those RIOs that are based on founding contracts containing open-ended commitments.

Ultimately, our findings suggest that the creation and subsequent institutional evolution of the EU has made a difference to the evolution of institutions in RIOs elsewhere; counterfactually, member states would have delegated less power to independent regional institutions in the absence of the EU. From this perspective, the analysis indicates that most existing theories of regionalism are incomplete because they neglect the interdependence that exists between different RIOs — and specifically the direct and indirect interactions occurring between prominent institutional pioneers and other regional organizations. More broadly, the analysis (re-)emphasizes two important insights that have been lost from view in many of the recent quantitative studies of diffusion, namely, that organizational pioneers are important providers of institutional designs for other RIOs and that diffusion processes are often hierarchically structured.

The article proceeds in four further parts. In the next one, we present our theory and hypotheses. Then, we operationalize the key variables and describe the data to be used in the analysis. The third part regards our estimation techniques and results. A final part concludes and discusses the argument’s theoretical implications.

## Theory and hypotheses

This article examines the argument that the EU systematically conditions processes of institution building within other RIOs. We first theorize two pathways of EU influence, drawing on the analytical distinction between active and passive forms thereof, before turning to the conditions under which they are likely to become salient within a particular organization.

### Pathways of EU influence

The first pathway captures the EU’s *active* influence on other RIOs. Here, EU influence is the result of activities consciously designed by a range of EU actors — being aimed at actively shaping institution-building processes elsewhere. Supporting regional integration has been a declared goal of the EU ever since the early 1970s.^2^ Apart from a few global powers and selective ‘strategic partners’ with which it has started to engage in the last decade or so, the EU regularly deals with non-member countries on a group-to-group basis rather than bilaterally. Over time, it has developed a holistic policy to support institution building within many RIOs that includes technical and financial assistance, the negotiation of cooperation and trade agreements, as well as political dialogue. This support is a distinctive feature of the EU’s external relations, and it is driven by a self-interested desire to generate economies of scale in foreign markets (Robles, 2008) — as well as constituting an attempt to ‘lay down an identity marker’ (Grugel, 2004: 621).

One way in which the EU’s active support affects institutional evolution within other RIOs is through the incentives offered. Through direct engagement, the EU may change ‘the relative size of payoffs associated with [institutional] alternatives’ (Braun and Gilardi, 2006: 310; see also, Simmons et al., 2006). Sociological institutional scholars refer to this mechanism as ‘coercive isomorphism’, defined as ‘both formal and informal pressures exerted on organizations by other organizations upon which they are dependent’ (DiMaggio and Powell, 1983: 150). The EU applies pressure through both financial incentives and institutional engagement. Financial incentives can be both positive — the strengthening of regional institutions can result from a desire to attract EU funding — and negative —institutional change is the result of a desire to avoid existing resources being withdrawn (Schimmelfennig and Sedelmeier, 2005). Even though the EU does not tie the provision of financial support directly to particular types of institutional change occurring, financial dependence on the EU can powerfully affect institution-building processes. Moreover, the institutional engagement of the EU with other RIOs may induce institutional feedback effects — a key insight of the literature on institutional overlap and regime complexity, as well as of that on organizations (DiMaggio and Powell, 1983; Gómez-Mera, 2016). Structured interaction with the EU requires counterparts to create mechanisms for such coordination, which may, in turn, have knock-on effects for institution building in the RIO in question itself.

The case-study literature provides evidence for the existence of this mechanism. EU threats to withdraw funding have previously provided a powerful impetus for institutional reform in the Southern African Development Community (SADC), an organization that is highly dependent on external donor funding. When the EU and other donors (mainly EU member states) considered shifting their financial support to other RIOs in the mid-1990s, this ‘threat’ catalysed an organizational restructuring that entailed, *inter alia*, a strengthening of the Secretariat and the establishment of a European Court of Justice-type SADC Tribunal (Gray, 2014b; Lenz, 2012: 163–64, 166).

Another way in which active EU influence affects regional institution building elsewhere is through ‘socialization’, which can be defined as a ‘process of interaction that involves changing attitudes about cause and effect in the absence of overt coercion’ (Checkel, 2001: 562). Relying on instrumental and constructivist assumptions, direct interaction between actors creates channels for communication that provide opportunities for teaching and persuasion (for an overview, see Checkel, 2005). Through interaction with the EU, RIO policymakers receive relevant information not only about what (and how) institutions work in the EU, but also about salient institutional developments in the process of European integration. Most immediately, such interaction might help problematize the institutional status quo in an RIO — as well as frame the way in which the problem is understood, and what potential solutions to it might look like (Duina and Lenz, 2016; Finnemore, 1993). Over time, such interaction may further lead to the generation of common knowledge about ‘good’ institutional solutions to particular problems (Grobe, 2010). Research into bounded rationality and decision-making has shown that policymakers often learn from information that is readily available (Meseguer, 2006), a condition that opportunities for direct communication positively affect. Similarly, socialization research shows that the intensity and duration of contact crucially shapes the extent of adoption of new ideas about cause and effect (Bearce and Bondanella, 2007; Checkel, 2005).

The literature has documented a wide degree of EU diffusion in the realm of regional courts (see Alter, 2012). The creation of the Andean Court of Justice in 1979, modelled on the European Court of Justice, is a pertinent example of active EU influence that occurred ‘not as a result of direct pressure from or financial linkages to the EU’ (Gray, 2014b: 19). Instead, networks of Andean and EU experts in the legal realms, which served as settings for learning and persuasion, played a key role in the aforementioned institution’s establishment. Saldías (2013) offers a detailed account of how personal connections between EU legal experts and influential consultants, as well as officials, in the Andean region led to a change in their beliefs about cause–effect relationships regarding effective legal systems within economic integration schemes.

Even though theoretically distinct, the incentive- and socialization-based mechanisms of EU influence often operate together in real-world situations. Thus, we treat them as complementary and mutually reinforcing ways by which active EU engagement with other RIOs affects the latter’s institutional evolution, suggesting the following testable proposition:

Hypothesis 1 (Active EU influence): The more extensive active EU engagement with other RIOs is, the more likely they are, *ceteris paribus*, to develop stronger regional institutions.

The second pathway captures the EU’s *passive* influence on other RIOs. The EU is the most successful pioneer of institutionalized economic cooperation between neighbouring countries in the post-Second World War era. This pathway, then, captures the idea that EU influence stems from the success and attractiveness of its institutional designs.

One way in which the EU’s own institutional evolution shapes regional institution building elsewhere is through learning. Learning as a mechanism of diffusion is concerned with information about the effects generated by the institutional choices of others, and often by those that pioneer them (Gilardi, 2012; Simmons and Elkins, 2004; Simmons et al., 2006). As institutional innovations start to take effect, they allow other policymakers to gauge whether they are successful in generating the desired outcomes. Familiarity and success are thus key conditions for learning. As Ovodenko and Keohane (2012: 523) note, ‘institutional designs that are familiar and perceived by a wide variety of participants in negotiations as successful in relevant contexts should have greater chances of being adopted’. Moreover, diffusion is facilitated by theorization, whereby cause–effect relationships derived from a specific experience become theorized as being generally applicable. By abstracting from the specificity of the context in which desirable effects are initially generated, theorization suggests that ‘similar practices can be adopted by all members of a theoretically defined population, with similar effect’ (Strang and Meyer, 1993: 496). Institutional pioneers, then, may shape decision-making abroad by providing new information that others can learn from.

From this perspective, the EU’s own institutional evolution can be expected to have affected regional institution building elsewhere. Many policymakers in other regions are familiar with EU institutions. Recent research on outside perceptions of the EU indicates that political elites in countries involved in regional integration processes view the EU not only as successful, but also as highly relevant to their own efforts (Chaban et al., 2009). As EU institutions evolve, the information derived from the EU experience is also likely to evolve. Similarly, the EU is the most theorized RIO, and such theorization is also likely to evolve over time — tracking the EU’s own institutional development. For instance, the widely recognized insight that allowing individuals to access regional courts is key to the effectiveness of a regional legal system could only develop after individuals had started to regularly use the EU one; this took decades to emerge, and was a connection forcefully established by Alter’s work (for an overview, see Alter, 2014). In this vein, Jetschke and Murray (2012) detail in a case study of the Association of Southeast Asian Nations (ASEAN) how learning from the EU shaped the former’s institutional reform process in the 2000s.

Emulation is another way in which institutional pioneers might passively affect institutional evolution elsewhere. This phenomenon is concerned with the social construction of appropriate behaviour, and can be defined as a process whereby ‘actors model their behaviour on the examples provided by others’ (Lee and Strang, 2006: 889). As organizational fields become structured through associational processes, they develop standards for the legitimate institutional forms that organizations gradually adopt in an attempt to enhance their legitimacy — and ultimately their chances of long-term survival (DiMaggio and Powell, 1983). Institutional pioneers are particularly likely to be emulated given the premium that exists in organizational fields for appearing similar in structural form to the most admired and successful organizations (Haveman, 1993). Drastic change in one such organization can therefore induce similar change in other organizations. As Weyland (2008: 290) notes for the sources of domestic policy change, ‘drastic change in one country often prompts emulation efforts in other nations by calling attention to problems and offering ideas for solutions’.

From this perspective, the EU’s own institutional evolution can be expected to have affected regional institution building elsewhere. The EU is widely seen as ‘the most advanced model of regional integration [in the world]’ (Jetschke and Murray, 2012: 185). Hence, it is plausible to posit that the EU is the main exemplar among those RIOs whose behaviour is likely to be emulated by others. EU institutions might not only provide boilerplate solutions to given problems; institutional change in the EU may also alert regional policymakers to the urgency of addressing certain problems inherent in regional integration in the first place (Duina and Lenz, 2016). It should be noted that positing emulation as a mechanism of EU influence does not necessarily imply that the wholesale adoption of EU designs by other RIOs is a given. It is also conceivable that individual member states emulate such models and put them forth as a bargaining position in the course of institutional negotiations. To the extent that bargaining outcomes reflect more than lowest-common-denominator decisions — an assumption that appears reasonable under conditions of iterative bargaining and geographic ‘lock-in’ — then such emulation would nudge the overall outcome towards the emergence of stronger regional institutions.

Processes of institutional emulation of the EU are well documented in the case-study literature. In a recent article on ASEAN, Rüland (2014: 246) suggests that it ‘mimicked European structures of interest representation’ in the 1970s in an attempt to regain legitimacy and enhance the organization’s survival prospects. Similarly, Jetschke (2009) portrays ASEAN as an ‘isomorphic institution’ that has continuously emulated EU institutions as a way to enhance its legitimacy. She demonstrates a striking temporal coincidence between institutional change in the EU and similar institutional changes in ASEAN, which is suggestive of a form of passive EU influence — occurring due to ASEAN’s desire to appear legitimate in the eyes of important both internal and external audiences.

These arguments lead to a second hypothesis concerning the EU’s passive influence on other RIOs:

Hypothesis 2 (Passive EU influence): As the EU enhances its institutional authority over time, other RIOs are, *ceteris paribus*, more likely to build stronger regional institutions.

### Scope condition of EU influence

Under what conditions are the identified two pathways likely to become salient in affecting processes of institution building beyond the EU’s borders? Diffusion studies conventionally treat internal determinants of institutional and policy change as mere controls, or null hypotheses, to demonstrate that diffusion does indeed matter (Simmons et al., 2006). Nevertheless, most diffusion scholars recognize that these two sets of factors often *interact* in generating outcomes. Lee and Strang (2006: 888) state, for example, that ‘international influences are integrally connected to national politics’, while Checkel (2001: 553) argues that ‘domestic politics — in particular, institutional and historical contexts — delimit the causal role of [norm diffusion through] persuasion/social learning’.

Recent research has sought to model this interaction (Grigorescu, 2010). The theoretical reason for such internal–external interplay is well established, and has been succinctly stated by early scholars of the ‘second image reversed’ perspective. As Gourevitch (1978: 911) notes, even compelling external pressures ‘are unlikely to be fully determining, save for the case of outright occupation. Some leeway of response to pressure is always possible, at least conceptually’. The analytical challenge, then, is to identify those organizational structures that determine the influence of external pressure.

We posit that contractual incompleteness is a key organizational characteristic conditioning the degree of EU influence. It is increasingly recognized that international organizations vary in their ability to adapt to changing circumstances, including pressures of diffusion. Not all international organizations are, as International Relations scholars widely assume, ‘notoriously resistant to reform or redirection’ (Barnett and Finnemore, 2004: 2). Instead, some of them regularly engage in institutional change. We argue that such variance is a function of the degree of completeness of the contracts upon which they are based. The existence of any international organization rests upon a contract in which states voluntarily agree to bind themselves to a set of formal rules to facilitate cooperation. While all such contracts are incomplete to some degree, they nevertheless vary in the extent to which they contain open-ended commitments. At one extreme, founding contracts are fixed — with cooperation being specifically geared towards achieving some *pre-defined* and concrete result, such as establishing a free trade area. At the other extreme, contracts are open-ended in that the ultimate intended result of cooperation is only vague and ill-defined. In this case, cooperation is intended to evolve over time in ways that cannot be conceived of from the outset; the *process* of cooperation has intrinsic value because the result is largely indeterminate.

Consider two contrasting examples: the North American Free Trade Agreement (NAFTA) approximates a fixed contract. The final goal of the cooperation ‘process’ is to create a free trade area with a pre-defined scope that entails mainly free trade in goods, services and investment. Once this is achieved, the organization will have fulfilled its purpose; NAFTA is not intended to be an organization that develops further thereafter. The Andean Community (CAN), in contrast, rests on an open-ended contract behind which the ultimate ambition is to create a ‘homogeneous society’ in the Andes. This is an ill-defined purpose that is impossible, and unfeasible, to define in terms of the steps needing to be taken to that end from the very outset. Thus, in this case, the process is expected to evolve over time.

Our understanding of contractual incompleteness as open-ended commitments thereby differs from the more conventional understanding, which captures the extent to which contracts ‘specify the full array of responsibilities and obligations of the contracting parties, as well as anticipate every future contingency that may arise throughout the course of the exchange relationship’ (Cooley and Spruyt, 2009: 8). This conventional understanding refers to the specificity of commitments in ‘existing’ policy areas. Crucially, these two contractual characteristics do not necessarily co-vary. Even organizations based on open-ended commitments initiate cooperation in specific policy areas, for which the commitments required might be relatively detailed. The founding contract of the Organization of Eastern Caribbean States, for example, contains open-ended commitments regarding the ultimate purpose of the organization, but it also contains specific policy ones regarding the creation of a common market — these commitments are rather detailed, together encompassing almost 30 pages of text. Importantly, the creation of a common market is only seen as an initial step in a longer journey towards ‘closer union among the peoples of the East Caribbean’.

These differences in contractual completeness matter, as a voluminous body of literature in institutional economics has theorized. Incomplete contracts have the virtue of being flexible in the face of exogenous shocks and of being apposite when there is uncertainty about the nature of the good or service to be provided. They endow participants with an enhanced capacity for adaptation because they entail open-ended commitments that can be adjusted to unforeseen circumstances (Gibbons and Henderson, 2012; Hart and Moore, 2008). Such open-ended cooperation projects resemble nation-building processes in their absence of an overarching master plan. The inherent flexibility of incomplete contracts means that institutions, which structure cooperation on substantive commitments, tend to evolve as commitments change, and thus become specified over time.

Institutions, in this case, not only serve to lower transaction costs by structuring and enforcing cooperation (Abbott and Snidal, 1998; Keohane, 1984); they also play an important role in ‘discovering’ an evolving process of cooperation (Marks et al., 2014). Fixed contracts, on the other hand, are bound to be inherently static. Their purpose is precisely to engage in a well-specified range of activities that are detailed *ex ante*. Institutions can, in this context, be designed at the outset, and they are less susceptible to external pressure. This leads to a third, conditional hypothesis about EU influence:

Hypothesis 3 (Conditional EU influence): The higher the contractual incompleteness of an RIO, the more likely it is that EU influence — both active and passive — will lead to stronger regional institutions emerging.

## Operationalization of variables and data

To test our hypotheses, we analyse the process of institution building in 34 RIOs from 1950 or the year of their establishment to 2010. We define an RIO in conventional terms as a formal international organization composed of three or more geographically proximate states having a continuous institutional framework. RIOs, then, are conceptually distinct from agreements such as the EU–Mercosur Interregional Framework Cooperation Agreement, alliances such as the Cairns Group and informal arrangements such as ASEAN+3 in their formality. RIOs are based on a written contract formally entered into by their member states, that is, they are designed for a continuous purpose and therefore have a capacity for ongoing collective decision-making. This involves, at the very least, a permanent and independent bureaucracy and a standing decision-making body.

In compiling the sample, we consulted the Correlates of War data set and selected organizations that have a distinct physical location or website, a formal structure (i.e. a legislative body, executive and administration), at least 50 permanent staff (based on information in the Yearbook of International Organizations), a written constitution or convention, and a decision-making body that meets at least once a year. We identified 34 RIOs (including the EU itself) that fit all or all but one of these criteria, including two RIOs that no longer exist — the Council for Mutual Economic Assistance and the first East African Community (EAC). The sample (listed in online Appendix A) is broadly comprehensive of states and continents, and includes all RIOs that have exercised significant authority in the years since 1950.

We see two reasons for limiting the sample to RIOs that have standing in international politics. The first is practical. The data requirements for a test of the proposed hypotheses would involve evaluating RIOs on the basis of significantly more information than was available in any prior data set; thus, given time and financial constraints, it makes sense to focus on those RIOs that have left some footprints in the primary sources. In most cases, they also feature in the secondary literature, hence our decision to exclude RIOs that have no website, address or that are poorly staffed. Second, while we think our argument might apply broadly, we suspect that states — including EU actors — would overall be more likely to pay attention to those RIOs that have a baseline level of financial resources available to them.

### Dependent variable

At the heart of this study is the strength of regional institutions, and their evolution over time. Our very definition of RIOs eliminates substantial variation in basic institutional features. All or almost all of the organizations in our sample have the following: an independent secretariat possessing administrative functions; a standing body, generally composed of national ministers, that regularly convenes in order to adopt secondary legislation; and some form of executive organ that supervises implementation. The vast majority of RIOs also feature some form of dispute settlement body, and one comprised of non-state actors such as parliamentarians, business representatives and/or non-governmental organizations. Whereas these features distinguish RIOs from other international institutional frameworks such as agreements, alliances or informal arrangements, they are insufficient to capture meaningful variation in institutional strength *within* this group. Therefore, we need to move beyond such basic measures of institutionalization to gauge variation in regional institution building — and the EU’s specific influence on it.

One way to do this is to construct a more fine-grained measure of institutional design that not only codes the existence/non-existence of important institutional actors, but also seeks to estimate their competences in decision-making. Our measure focuses on those institutional actors who enjoy some degree of independence from member state control, and who thereby characterize ‘supranational’ elements in RIOs. Such independence is often conceptualized as delegation, which can be defined as ‘a conditional grant of authority from a principal to an agent that empowers the latter to act on behalf of the former’ (Hawkins et al., 2006: 7; for an overview of relevant concepts, see Hooghe and Marks, 2015). The principals — meaning the member states — retain ultimate control, but delegated agents enjoy a degree of autonomy that can, and often does, change over time. Delegation is considered to be of major theoretical significance, and is widely used in other empirical studies of RIOs, preferential trade agreements and global organizations. It is also a hard case for assessing EU influence because delegation entails sovereignty costs. By empowering independent agents, member states lose full control of the process of regional cooperation; they also enhance the danger of unanticipated consequences occurring due to agency slack.

The dependent variable *Delegation* is an additive index of formal delegation in decision-making processes for each year of a given RIO’s existence (Hooghe et al., forthcoming). It measures the extent to which member states empower third parties to adjudicate disputes, provide expert information, select or prioritize proposals, and, at the authoritative extreme, propose policy initiatives, make binding decisions and/or penalize contract violations. The extent of delegation is a function of: (1) the composition of the organized bodies within a given regional organization (general secretariat, assemblies, executives, judicial bodies, consultative bodies) with respect to their independence from member state control; and (2) the authoritative competencies of regional bodies in agenda setting, final decision-making and adjudication in (3) one or more of six possible decision areas — accession, suspension, constitutional reform, budgetary allocation, financial non-compliance and policymaking (for further detail, see online Appendix B). The measure assesses the formal rules that can be observed in treaties, constitutions, conventions, special statutes, protocols and rules of procedure, which has a distinct advantage in that they can be specified independent of actual behaviour.

**Figure 1.**
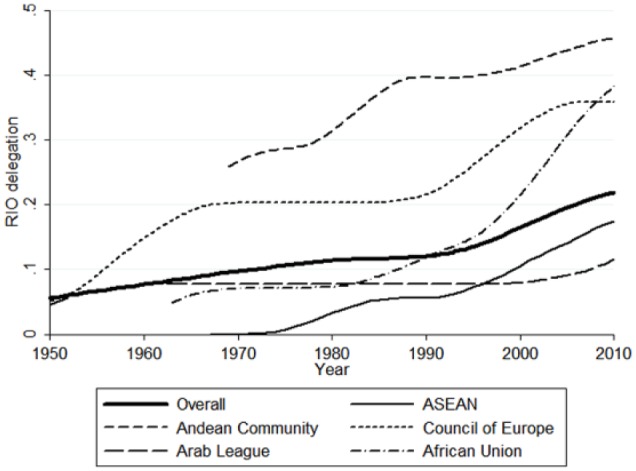
Delegation in selected RIOs, 1950–2010.

Figure 1 gives a sense of the sample variation in *Delegation*, and shows its overall evolution (black line) over the period 1950–2010. We see that delegation within RIOs has gradually increased over time, with a particularly marked rise coming after the end of the Cold War. Concerning variation across RIOs, median values range from zero for the South Asian Association for Regional Cooperation and the Southern Africa Customs Union to 0.43 for the second East African Community (EAC), with the remaining regional organizations being distributed fairly evenly in between. Three organizations (Council of Europe, EAC, Nordic Council) have a parliamentary body that operates as a non-state assembly. Four organizations (Central African Economic and Monetary Union (CEMAC), Economic Community of Central African States, Economic Community of West African States (ECOWAS), and SADC) have a general secretariat with an exclusive right of initiative. Two organizations (CEMAC, Common Market for Eastern and Southern Africa) feature a supranational court that provides access to non-state actors and passes preliminary rulings, and whose rules have direct effect. In the lowest third of the data set are regional organizations as diverse as Asia-Pacific Economic Cooperation (APEC), the Gulf Cooperation Council and the Latin American Integration Association. These organizations have little more than a weak general secretariat that, at most, draws up the budget or has a non-exclusive right to initiate policymaking. Although none of the organizations in the sample have achieved the level of delegation reached by the EU, some have gradually evolved in this direction — while others remain at low levels thereof.

### Independent variables

Our two key independent variables are active and passive EU influence, respectively. We operationalize *active EU influence* through an aggregate index that measures the EU’s institutionalized engagement with other RIOs. In the absence of prior measures, we construct an index of EU engagement that consists of three components designed to capture the various ways in which active EU influence affects regional institution building elsewhere, which we describe briefly in the following (for further detail on these three components, see online Appendix C). The index is quantitative, and the components are normalized and weighted equally in the aggregate index. The Cronbach’s alpha is 0.863, which indicates high scalability. We use the index in our main statistical analysis because it allows us to capture the distinct logics separately while also enhancing the robustness of our measures. However, we employ its individual components as robustness checks. The expectation is that more intensive engagement with the EU will lead to more delegation occurring in RIOs elsewhere.

The first of the three components is the EU’s financial support to other RIOs, which is the main way in which the EU shapes incentives for institutional change. EU funding is an ordinal variable (with four categories) that captures the amount of funding directed to a specific RIO in a given year, encompassing both institutional and project support (x < €1 million; €1 million < x < €4 million; €4 million < x < €8 million; x > €8 million). Funding streams were coded on the basis of a variety of official documents, primarily issued by the EU itself, and we augmented these data with information from the RIOs themselves. About half of the organizations have not received any funding from the EU during their lifespan.

The second component is EU interregional cooperation agreements, a more indirect way by which active EU influence shapes incentives for institutional change. Insti-tutionalized cooperation measures the policy scope and obligation of all of the EU’s agreements with other RIOs in our data set based upon the assumption that interregional agreements with a wider policy scope and a higher degree of obligation are more likely to exert stronger effects of institutional feedback. We assess an interregional agreement’s policy scope based on a list of 29 policy areas, one adapted from Lenz et al. (2015). The binding nature of cooperation is assessed on a three-point scale that measures the nature of obligations associated with cooperation (non-binding versus binding), as well as the existence of interregional monitoring institutions — which forms the highest category of obligation. Our institutionalized cooperation score is the sum of the two standardized components of policy scope and obligation. We assess a total of 15 agreements that, between them, involve nine different RIOs.

The third component is the frequency of institutionalized contact between the EU and an RIO, which is widely used as a proxy for opportunities for teaching and persuasion in quantitative studies (Bearce and Bondanella, 2007: 712–713). EU contact is a count of instances of institutionalized contact between EU representatives and their counterparts in a given year across three levels: (1) ministers and heads of state; (2) parliamentarians; and (3) technical experts, including representatives of the European Commission. The count assumes a value of 3 when all three sets of actors met in a given year, and 0 when none of them met — or when no institutionalization of contact took place. Contacts were coded on the basis of a variety of documents, such as meeting programmes, draft agendas or final communiqués of interregional meetings, augmented by website entries and written information elicited by email. The EU has had institutionalized contact with 22 out of the 34 organizations in our data set, but their frequency and intensity have varied strongly.

Our second key independent variable is *passive EU influence*. We expect the EU’s own institutional trajectory to affect the degree of delegation in other RIOs. We operationalize passive EU influence as the evolution of the level of delegation in the EU, coded in the same aforementioned way as we did for other RIOs. With the exception of the transition from the European Coal and Steel Community towards the European Economic Community in the years following 1957, EU delegation has increased over time. This is most markedly true between the Single European Act of 1986 and the 1999 Treaty of Amsterdam, after which delegation tapers off. As a robustness check, we use an alternative measure of passive EU influence calculated by Frank Schimmelfennig on the basis of Börzel’s (2005) conceptualization and measurement of the scope of European integration. This captures the involvement of supranational bodies and Council voting rules in EU decision-making in a given policy area (for details, see Börzel, 2005: 220–221). The two measures are highly correlated (0.88).

Finally, we operationalize the hypothesized scope condition of EU influence, namely, that it depends on an endogenous capacity for RIO institutional change. In the absence of prior measures, we take *contractual incompleteness* as a trichotomous variable that taps the extent to which the commitments that member states engage in are open-ended. There are two key dimensions of incompleteness, which we combine into a single indicator (see Table 1). The first one is the open-endedness of the policy scope, which we assess on the basis of the stipulated objectives of cooperation. This dimension distinguishes organizations with a concrete and narrow organizational objective (fixed) — for example, the Organization of Arab Petroleum Exporting Countries, whose principal aim lies in ‘the co-ordination and unification of the petroleum policies of Member Countries’ (Art. 2, 1968 OAPEC Agreement) — from those that pursue only a vague and broad-based objective vis-a-vis cooperation (open-ended) — such as the Shanghai Cooperation Organization, whose aim is ‘to consolidate multidisciplinary cooperation in the maintenance and strengthening of peace, security and stability in the region’ and to ‘facilitate comprehensive and balanced economic growth, social and cultural development in the region through joint action on the basis of equal partnership’ (Art. 1, 2002 SCO Charter).

**Table 1.** Measuring contractual incompleteness.

	Policy scope	Actor scope
***High***	Open-ended	Open-ended
***Medium***	Open-ended	Fixed
***Low***	Fixed	Fixed

The second key dimension is that of the open-endedness of actor scope, which we assess on the basis of whether the treaties emphasize national sovereignty and make reference to governments, member states or countries as the *only* relevant actors. It distinguishes state-centred organizations (fixed) from those that provide for the potential participation of a wider group of actors in the cooperation process (open-ended). State-centred organizations achieve cooperation objectives through inter*governmental* cooperation, wherein national governments are the only legitimate actors. This is reflected in treaties regularly emphasizing national sovereignty and making continuous reference to governments, member states or countries as the *only* relevant actors. Organizations with a more open-ended actor scope, in contrast, do not have these characteristics. Actors encompass loosely defined representatives of ‘the people’, as well as national governments. Such organizations typically include transformational commitments vis-a-vis their societies, which are expressed in references made to a ‘union of peoples’, ‘community of peoples’ or ‘ever closer union’.

At the point in time of their founding, the 34 regional organizations in our sample divide in roughly equal parts across the three categories identified in Table 1. While an organization can change its degree of contractual incompleteness over time, this is quite rare. We code six single interval moves towards greater incompleteness. Our analysis, presented in the following, uses the original contracts for the purpose of this investigation.

### Control variables

Our argument about EU influence challenges two other sets of arguments about the drivers of regional institution building, ones that we control for in the analysis. First, our argument implies that EU influence is not reducible to other types of external influence operating beyond the confines of individual RIOs, including processes of institutional diffusion not related to the EU.

#### Regional delegation

The diffusion literature suggests that patterns of diffusion cluster among neighbouring countries or adjacent regional organizations, that is, there is a strong geographic element to diffusion processes (Weyland, 2008). This ‘neighbourhood effect’ may result from contact and exchange, the sharing of important cultural characteristics, and/or membership overlap. In order to capture such effects, we measure *Regional delegation* as the average level of delegation of all neighbouring RIOs in the same geographic region (either the Americas, Europe, Africa, the Middle East or Asia), with the exception of the RIO in question itself.

#### Global delegation

We also control for genuinely global rather than regional diffusion processes. Sociological institutionalists, in particular, expect RIOs, as a distinct category of organization, to become more similar in their institutional structure over time due to the emergence of a norm of ‘acceptable’ levels of delegation (Powers and Goertz, 2011). We tap into such processes by measuring in a given year the average level of delegation in the sample, with, again, the exception of the specific RIO in question.

#### Globalization

The ‘New Regionalism’ literature, in particular, emphasizes globalization as being a major driver of regionalism in its various forms (for an overview, see Söderbaum and Shaw, 2003). As economic, social and political exchanges that cross national borders grow, a variety of actors are likely to engage in cooperative endeavours in order to manage such interdependencies. The delegation of competences to RIOs by state governments might also follow this logic. We divulge the impact of *Globalization* through the widely used KOF Index of Globalization, which captures the economic, social and political connections that countries have with the rest of the world (1970–2010) (Dreher, 2006). We include an aggregated measure based on the RIO mean of each member state’s globalization score for a given year.

#### Cold War

Various other developments that we might expect to affect RIO delegation cluster at the end of the Cold War, and we introduce a time dummy (0 = post-1990) to nullify their potential confounding effects. The end of intense ideologically driven bipolar competition created new demands for regional cooperation, ones that might be reflected in deeper institutionalization. The end of the Cold War also roughly coincides with a stalemate in the Uruguay Round of multilateral trade negotiations under the General Agreement on Tariffs and Trade. As the prospects for continued multilateral trade liberalization appeared bleak by the late 1980s, states turned towards regional options instead — a development that may have led to (the creation of) institutionally more ambitious RIOs.

Beyond alternative external influences, our argument about EU diffusion challenges, above all, explanations that locate the drivers of institution building in *intra*regional dynamics. The structural characteristics of units are typically the null hypothesis of diffusion studies. We consider several controls internal to each RIO, including the most important explanations for international institutional change in general.

#### Intraregional trade

Perhaps the most firmly grounded expectation in the literature on international institution building is that it should co-vary with economic interdependence (Haftel, 2013; Keohane, 1984). Economic exchange develops its welfare-improving potential to the fullest with stable, predictable property rights. Hence, trade that traverses international borders creates a demand for coordination among states so as to provide uniform rules. Reducing barriers to cross-border trade is a core rationale of many RIOs, and one might expect, therefore, that the growth of trade interdependence within a regional organization leads to greater delegation. We measure trade interdependence, *Intra-RIO trade*, as a region’s total trade (imports plus exports) as a proportion of member countries’ total trade.

#### Power asymmetry

Scholars in the tradition of Waltzian neorealism hypothesize that powerful states reject strong institutionalization because it inhibits unilateral action, and instead prefer intergovernmental arrangements (Abbott and Snidal, 2000: 448). Conversely, hegemonic stability theory suggests that an unequal distribution of power may expedite the provision of public goods and a hegemon may find the rule of law useful in eliciting the compliance of weaker members (Krasner, 1976). We control for these possibilities with a measure of power dispersion, *Power asymmetry*, being the ratio of the material capabilities of the most powerful member state to the average of all other members. The Composite Index of National Material Capabilities (CINC) Version 4.0 provides a summary measure of military expenditure, military personnel, energy consumption, iron and steel production, urban population, and total population for individual countries (Singer, 1988).

#### Members

The extent of delegation within an organization might be sensitive to the size of its overall membership base. As the number of members grows, decentralized cooperation in the absence of delegated institutions may become more costly as a result of issue cycling and increasing informational asymmetry (Hawkins et al., 2006; Koremenos et al., 2001: 789). We measure *Members* as the natural log of the absolute number of member states in a given year, assuming that the effect of one additional member joining declines as the absolute number increases.

#### Democracy

Norms of appropriate behaviour in democratic states, or alternatively the political context in newly democratizing countries, may render elites more willing to delegate to international organizations (Grigorescu, 2015). An implication of the findings of the democratic peace literature is that autocracies are more likely than democracies to be fearful of exploitation. Newly democratizing states, in particular, may use international institutions as external commitment devices (Moravcsik, 2000). We measure *Democracy* as the annual Combined Polity Score in the Polity IV data set.

#### Per capita gross domestic product

Finally, we control for the mean per capita gross domestic product (GDP) of member states in an RIO in a given year on the premise that the richer the members, the greater the demand for international cooperation — and, correspondingly, the degree of delegation to international organizations. (Summary statistics and bivariate correlations for all variables used in the analysis are contained in online Appendix D.)

## Estimation and results

We now turn to the empirical testing of our claim that the EU systematically shapes processes of institution building in other RIOs. We first discuss issues of model specification, before then turning to the results and their robustness. A discussion of the control variables follows thereafter.

### Model specification

The dependent variable is the level of delegation in an RIO, expressed as a continuous variable ranging from 0 to a theoretical maximum of 1, though the highest value in our data set is 0.452 (achieved by the Andean Community in 2006). As noted, our data set examines 34 RIOs between 1950 and 2010. Thus, our analysis is of panel data, meaning data that vary across time for a number of entities — that is, RIOs. Since we have only one dependent variable, we select linear least-squares regression as our technique. It must be noted that RIOs come into being at different times, meaning that our data are unbalanced; hence, we use fixed-effect models with robust standard errors (Greene, 2008). Meanwhile, *RIO delegation* is lagged by one year in order to ensure that we mitigate any issues that may arise from endogeneity.

Furthermore, contractual open-endedness is a key variable, which takes two forms: analysed both on its own and as part of an interaction with active and passive EU influence. Contractual open-endedness takes values of 1 (fixed contract), 2 (intermediate contract) and 3 (open-ended contract). As an interaction term, it serves to uncover whether active and passive EU influence vary across RIOs that differ as regards their contractual characteristics. The interaction takes the form:

$$Y\;=\;c\;+\;ax_{1}+\;bx_{2}+\;d\left(x_{1}\;x_{2}\right)\;+\;SE$$

where *x_1_* is the continuous variable *active EU influence/passive EU influence, x_2_* is the factor variable *contractual open-endedness* and *d(x_1_x_2_)* is the interaction term. In Models 3, 5 and 7, contractual incompleteness is used as a factor variable with a base of 1 (indicating, as noted, a fixed contract).

### Results

Results are presented in Table 2. The models are: (1) contractual incompleteness; (2) active EU influence; (3) active EU influence interacted with contractual incompleteness; (4) passive EU influence; (5) passive EU influence interacted with contractual incompleteness; (6) active and passive EU influence; and (7) active and passive EU influence interacted with contractual incompleteness.

**Table 2.** EU influence and delegation in RIOs.

	(1)	(2)	(3)	(4)	(5)	(6)	(7)
Contractual incompleteness	1.3^***^ (0.266)						
Active EU influence		0.848^***^ (0.285)	0.498^***^ (0.126)			0.67^**^ (0.299)	0.403^**^ (0.164)
* Intermediate contract			−0.134 (1.088)				−0.037 (0.293)
* Open-ended contract			0.587^*^ (0.346)				1.463^**^ (0.693)
Passive EU influence				6.044^***^ (1.220)	3.96^***^ (1.287)	4.785^***^ (1.208)	3.185^**^ (1.366)
* Intermediate contract					0.934 (0.791)		0.174 (0.618)
* Open-ended contract					3.323^***^ (0.861)		3.752^***^ (0.974)
Regional delegation	−0.308 (0.191)	−0.081 (0.211)	−0.117 (0.199)	−0.151 (0.245)	−0.172 (0.198)	−0.046 (0.221)	−0.158 (0.194)
Global delegation	4.881^**^ (2.297)	4.756^*^ (2.642)	1.319 (2.139)	0.463 (2.263)	−3.135 (2.094)	0.428 (2.226)	−3.106 (2.115)
RIO globalization	−0.004 (0.013)	−0.003 (0.013)	−0.016 (0.01)	0.008 (0.011)	−0.01 (0.009)	0.009 (0.011)	−0.005 (0.007)
Cold War	−0.103 (0.061)	−0.020 (0.080)	−0.086 (0.053)	−0.144^*^ (0.079)	−0.029 (0.046)	−0.146^*^ (0.082)	−0.034 (0.042)
Intra-RIO trade	0.010 (0.01)	0.011 (0.01)	0.013 (0.009)	0.009 (0.009)	0.01 (0.008)	0.009 (0.008)	0.008 (0.006)
Power asymmetry	0.001 (0.041)	0.03 (0.045)	0.016 (0.043)	0.003 (0.044)	−0.022 (0.038)	0.013 (0.042)	−0.011 (0.037)
Members	0.019^*^ (0.010)	0.015 (0.014)	0.013 (0.015)	0.016 (0.011)	0.012 (0.01)	0.013 (0.013)	0.008 (0.011)
Democracy	0.029 (0.023)	0.036 (0.034)	0.024 (0.024)	0.037 (0.031)	0.015 (0.017)	0.03 (0.033)	0.008 (0.016)
GDP	0.000^**^ (0.000)	0.000^*^ (0.000)	0.000 (0.000)	−0.000 (0.000)	−0.000 (0.000)	−0.000 (0.000)	−0.000 (0.000)
Constant	−5.58^***^ (0.595)	−3.26^***^ (0.652)	−2.92^***^ (0.428)	−6.1^***^ (0.81)	−5.33^***^ (0.644)	−5.5^***^ (0.889)	−4.91^***^ (0.593)
R2 — within	0.381	0.324	0.380	0.325	0.489	0.363	0.561
R2 — between	0.248	0.008	0.019	0.003	0.249	0.003	0.236
R2 — overall	0.170	0.072	0.106	0.057	0.220	0.092	0.198

*Notes*: All models use fixed effects and robust standard errors (in parentheses). ^*^ *p* < 0.1; ^**^ *p* < 0.05; ^***^ *p* < 0.01.

In line with Hypothesis 1, we find robust evidence that active EU influence — an aggregate index of funding, interregional agreements and institutionalized contacts — is associated with higher levels of delegation in other RIOs. This comes with positive and strongly statistically significant coefficients in Models 2 and 6. These results indicate that the EU’s efforts to boost regional institutionalization are successful, and do exert an independent effect on delegation. Disaggregating active EU influence into its three constituent components and conducting the regression with each of the components independently does not affect the results (for details, see online Appendix E.1).

Passive EU influence, on the other hand, is measured by the EU’s own delegation trajectory. Model 4 indicates that higher levels of delegation in other RIOs are associated with increases in the EU’s own delegated authority, and this result holds true when both active and passive EU influence are included (Model 6).

On its own, contractual incompleteness is significantly correlated with delegation — meaning that organizations based on more open-ended commitments are more likely to achieve high levels of delegation. This holds true for organizations with highly incomplete contracts when interacted with active and passive EU influence. These results are in line with our hypothesis that RIOs possessing an endogenous capacity for change are more likely to go through it.

The effect of active EU influence varies across different types of RIOs, as captured by contractual incompleteness. Specifically, active EU influence greatly increases in organizations that have an open-ended founding contract — as demonstrated by the positive and significant interactions in Models 3 and 7. On organizations that restrict the actors of cooperation to governments (intermediate contract) — let alone on those with a fixed contract — the EU has very limited influence. These results bolster our conditional hypothesis that active EU influence is most effective in RIOs that are disposed of an endogenous capacity for institutional change because unspecified commitments provide space for the creation of new institutional mechanisms — as well as the strengthening and reforming of existing mechanisms. Unsurprisingly, most of the case-study evidence that has demonstrated EU influence on other RIOs concerns organizations with open-ended founding contracts — including Mercosur, SADC and the CAN.

Just as for active EU influence, we find strong support for the idea that institutional evolution in the EU is associated with a change in delegation in specific types of organizations, namely, those based on open-ended contracts. Models 5 and 7 consistently show a positive and statistically significant effect for the respective interaction term. These results thus lend strong support to the conditional hypothesis that only organizations with open-ended commitments are responsive to changes in the EU’s own delegated authority. Where the ultimate purpose of cooperation is clearly defined from the outset, institutional change in an important reference organization — in this case, the EU — does not affect an organization’s own level of delegation.

**Figure 2.**
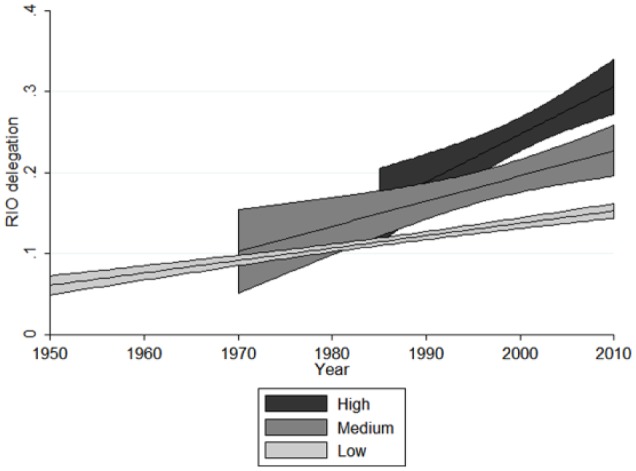
Active EU influence and RIO delegation for different levels of contractual incompleteness, 1950–2010.

How substantive is the effect of active engagement between the EU and other RIOs on delegation? Figure 2 plots the predicted values of RIO delegation for the different values (low, up to the 33rd percentile; medium, between the 33rd and 66th percentiles; high, between the 66th and 100th percentiles) of our active EU influence measure over time, with other variables being held at their mean. It shows that delegation grows faster the more closely the EU engages with other RIOs. The most pronounced effect is in RIOs deeply engaged with the EU; this is largely a post-Cold War phenomenon. Yet, even for RIOs that have medium-level engagement with the EU, the effect on delegation is noticeable, being statistically distinguishable from low or no active engagement from around 1980 onwards.

**Figure 3.**
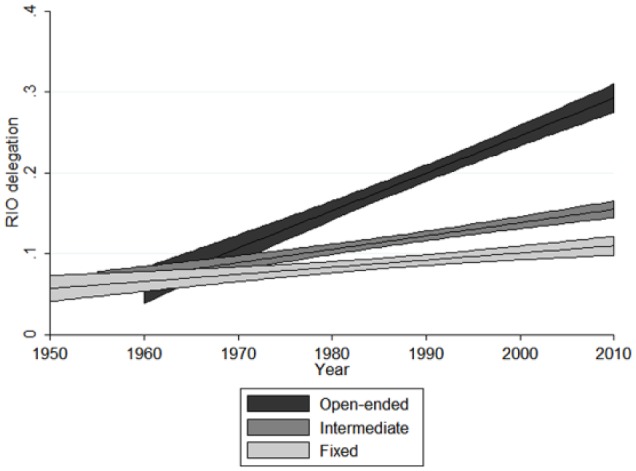
Passive EU influence and RIO delegation for different levels of contractual incompleteness, 1950–2010.

This is also the case for passive EU influence. Figure 3 plots the predicted effects of the interaction over time based on Model 5, with other variables being held at their mean. It illustrates that EU delegation exerts its strongest impact on RIOs with an open-ended founding contract. We interpret this result as evidence that the EU serves as an important reference point for learning and emulation processes, primarily for those RIOs that are similar to the EU with regard to open-ended commitments. Overall, these results lend strong support to the idea that, at least for specific types of organization, decision-making is indeed interdependent across organizations — and even in the absence of active EU influence.

Finally, our results also indicate that passive EU influence exerts a larger overall substantive effect on RIO delegation than active EU influence does. In concrete terms, an increase of one standard deviation in active EU influence leads to a 0.08 increase in delegation in other RIOs. This is equivalent to the establishment of a third-party dispute settlement body consisting of ad hoc arbitrators that, under certain conditions, can mandate retaliatory sanctions. A one standard deviation increase in passive EU influence, in contrast, is associated with an increase of 0.15 in delegation, almost twice as much, which translates into the establishment of a general secretariat with executive functions, budgetary competences and a non-exclusive right to set the agenda on policy — further to the creation of a parliamentary body with consultative functions. As expected, these effects are somewhat larger for RIOs with open-ended contracts. Here, a one standard deviation increase in active EU influence increases delegation by 0.1 — a value that grows to 0.18 for passive EU influence. However, one should keep in mind that the substantive effect of passive influence is spread over the entire lifetime of the EU and affects all organizations in our sample, whereas active influence targets individual organizations and varies in its intensity. As a result, sustained active EU influence can have effects comparable (and additional) to passive EU influence in specific organizations — such as in the case of ECOWAS, CAN or SADC.

### Robustness checks

A counter-argument may posit that active EU influence is endogenous to delegation because the EU might be interested in dealing with more established and significant organizations. In other words, high levels of RIO delegation may *attract* more extensive institutionalized engagement with the EU — rather than active EU influence causing an increase in RIO delegation per se. We therefore test for reverse causality by running the models of active EU influence with delegation as the independent variable, and active EU influence as the dependent variable — also including the interaction with contractual incompleteness. The results show that delegation is not a significant factor in determining whether the EU actively engages with a specific RIO or not (for details both on this and on all subsequent robustness tests, see online Appendix E.2).

On the basis of the assumption that channels of EU influence might be more informal due to membership overlap, we conducted the analysis while excluding European RIOs. However, this does not change the results already presented in Table 2. We also conducted the analysis using an alternative measure of passive EU influence drawn from Börzel’s (2005) study of the level and scope of European integration, having been updated by Frank Schimmelfennig for the recent period. This measure is highly correlated to our measure of passive influence (0.88). The results hold constant both for the alternative measure alone and in combination with active EU influence, but the interaction term loses statistical significance. Finally, there is no substantive change in results when we extend the lag on the EU influence variables to two or even four years.

### Controls

Finally, we consider the effects of our control variables. We first turn to alternative external influences. Regional diffusion appears to have a *dampening* effect (even though insignificant) on levels of delegation among neighbouring organizations in five continental macro-regions (the Americas, Europe, Africa, the Middle East and Asia), as indicated by the consistently negative sign of the coefficient. This represents a stark contrast to our finding that the direct and indirect pressures that the EU exerts on RIOs help to create stronger regional institutions, as it means that EU influence works in a different direction than trends in neighboring RIOs. Global delegation is volatile in its sign and does not appear to exert any systematic impact on RIO delegation. An ‘acceptable’ level of delegation appears not to have emerged among RIOs.

An RIO’s connectedness with the rest of the world (*RIO globalization*), which increased rapidly in tandem with the end of the Cold War, also seems to matter little. The sign changes across models, and it is never statistically significant. This is not surprising insofar as some of the most globalized RIOs — such as APEC, the European Free Trade Association and NAFTA — have low levels of delegation. Finally, the end of the Cold War had a positive influence on delegation in RIOs.^3^ This might have been due to the end of general bipolar competition, creating space for more independent RIOs, or specifically to the stalemate of multilateral trade negotiations, which led states to pursue alternative routes to trade liberalization. The fact that the Cold War dummy seldom reaches conventional levels of statistical significance suggests that other variables do pick up the ‘end of the Cold War’ effect.

We also find very limited support for all of the intraregional variables. Power asymmetry is not consistently signed, indicating that various configurations of power symmetry between members in terms of capabilities have no impact on delegation. This challenges the neorealist claim that powerful states are reluctant to cede sovereignty. We also find almost no support for the neoliberal-institutionalist claim that intraregional trade interdependence affects delegation, confirming a recent finding by Haftel (2013). Some of the strongest regional institutions can be found in regions where trade interdependence is comparatively low, such as Africa and Latin America.

There is some support for the rational design claim that more members lead to more delegation, confirming a finding by Hooghe and Marks (2015). The coefficients are consistently positive (but non-significant), suggesting that the functional pressures for delegation in large membership organizations often overcome the threat of decisional blockage that increases as the number of members goes up. Finally, there is some support for the influence of democracy. The coefficient is consistently positive, cautiously supporting the idea that RIOs with more democratic member states tend to delegate more extensively; however, these results are driven mainly by European RIOs. Finally, per capita GDP is irrelevant as a predictor of RIO delegation.

## Conclusion

In this article, we demonstrate statistically the active and passive influence of the EU on the trajectory of institution building in other RIOs, doing so by drawing on a new and original data set that measures variation in the institutional design of 34 organizations in the period from 1950 to 2010.

The findings of our analysis imply that existing theories of regionalism are incomplete, specifically because they focus on causal factors internal to a particular RIO and thereby neglect the wider contexts in which these organizations emerge and evolve. Even though some scholars acknowledge that RIOs change in response to external pressures — new regionalism scholars emphasize globalization, realists emphasize outside security threats — there is little recognition that institutional choices are regularly *inter*dependent between organizations. Further, institutional pioneers such as the EU have an incentive to actively shape other organizations in their field and, especially when successful, they serve as reference points by providing institutional designs that others can learn from and emulate.

This might be one reason why integration theories developed in the European context do not travel well. In the 1970s, neofunctionalists abandoned their endeavours to develop a general theory of regional integration, while liberal intergovernmentalism has barely been applied outside of Europe. Explanations for institutional innovations differ fundamentally from those for subsequent institutional adoption in diffusion research. Whereas the former tend to reflect the structural conditions of an organization — in other words, functional explanations are often pertinent — subsequent adoptions are often the result of diffusion — with endogenous conditions being insufficient explanations for a given institutional design (Finnemore, 1993). Hence, the relative weight of internal and external factors in the explanation of institutional evolution shifts over time towards the latter as institutional innovators become active proponents of their institutional design and an organizational field becomes structured. Existing theories of regionalism have largely neglected this insight.

Beyond theories of regionalism, our analysis also has implications for the quantitative literature on diffusion. The analysis shows that organizational pioneers, such as the EU, are important providers of institutional designs for other RIOs — thereby answering the seemingly simple question: where do the diffused institutional designs actually come from? Recent quantitative studies of policy and institutional diffusion have had a hard time answering this question because they generally conceptualize diffusion in terms of horizontal connections between units of analysis that are modelled through spatial lags, sometimes in a strictly dyadic set-up. The analytical concern therein is with identifying the relevant connections through which diffusion occurs (for an overview, see Gilardi, 2012). This approach has been powerful in establishing that policymaking is regularly interdependent between organizations, and also in identifying the relevant ‘reference groups’ that facilitate this diffusion. The current analysis implies, however, that such studies tend to misconceive of international diffusion processes as overly decentralized and uncoordinated. This article suggests that institutional diffusion can, in fact, also be an asymmetrical process, even a hierarchical one, in which influence primarily flows outwards from important institutional pioneers.

Beyond these broad theoretical implications, it is also worth asking whether the EU’s influence is likely to remain high for the foreseeable future — specifically in view of recent developments such as the Euro crisis, the migration crisis and Brexit. Scepticism is warranted as the EU’s credibility as the most successful RIO has suffered — thus also calling into question the attractiveness of the ‘EU model’. Consequently, EU policymakers’ enthusiasm to export that model may have waned too. Nevertheless, as long as the EU continues to provide financial support to regional institutions elsewhere and as long as stronger regional institutions serve important purposes for regional cooperation — a lesson from the EU experience that remains valid — its loss of influence may ultimately only be transitory. The strongest effects on EU influence may thus concern not the strength of regional institutions, but instead other important issues of regional cooperation that have been more directly affected by the various crises. Examples are the scope of membership or the ambition of integration objectives; for the foreseeable future, decisive moves towards economic and monetary union elsewhere are perhaps less likely.

## Notes

1. The relationship between design and actual cooperation in RIOs is ambiguous, and requires more systematic study. For contending views, see Haftel (2013) and Gray (2014a), who emphasize implementation gaps, and Alter (2014), who shows that institutional design makes a difference to cooperative outcomes.

2. For the sake of brevity, we use the acronym ‘EU’ to refer to both today’s EU as well as its predecessors.

3. It is negatively signed because it assumes a value of 0 after 1990.
